# Quantification of septal and whole slice myocardial blood flow by myocardial perfusion CMR is similar in healthy volunteers

**DOI:** 10.1186/1532-429X-16-S1-P12

**Published:** 2014-01-16

**Authors:** Bara Erhayiem, Ananth Kidambi, David P Ripley, Adam K McDiarmid, Steven Sourbron, John P Greenwood, Sven Plein

**Affiliations:** 1 Multidisciplinary Cardiovascular Research Centre & The Division of Cardiovascular and Diabetes Research, Leeds Institute of Genetics, Health & Therapeutics, University of Leeds, Leeds, West Yorkshire, UK; 2 Department of Medical Physics, University of Leeds, Leeds, West Yorkshire, UK

## Background

First pass myocardial perfusion CMR allows quantification of myocardial blood flow (MBF). MBF estimation with whole-heart tissue response may be useful in a variety of systemic diseases, but can be limited by suboptimal imaging in one or more segments. The interventricular septum (IVS) offers an attractive target for MBF imaging, as it offers higher signal and less partial volume artefact from blood pool. It has been proposed that T1 measurements taken from the IVS are more reliable than measurements from an entire short axis slice. We hypothesised that MBF estimation from the IVS would be similar to whole-heart estimation.

## Methods

Nine healthy volunteers underwent CMR at 3.0T (Philips Achieva TX, 32 channel receiver coil). First-pass perfusion imaging in three short-axis LV slices was performed during administration of 0.075 mmol/L/kg of gadobutrol at basal, mid-ventricular and apical short-axis slices. This protocol was performed following 3 minutes of 140 mcg/kg/min adenosine for stress perfusion and repeated 15 minutes later at rest. MBF estimation was performed using Fermi deconvolution (PMI v.0.4, [Sourbron, 2009]) with basal blood pool providing the arterial input. Tissue response with whole mid-ventricular myocardium and limited IVS contours were compared. Myocardial perfusion reserve (MPR) was calculated by dividing stress MBF by rest MBF. Adequate hemodynamic response was defined as heart rate increase ≥10/min or blood pressure decrease ≤10 mmHg or presence of significant chest discomfort or dyspnoea.

## Results

**Figure 1:**
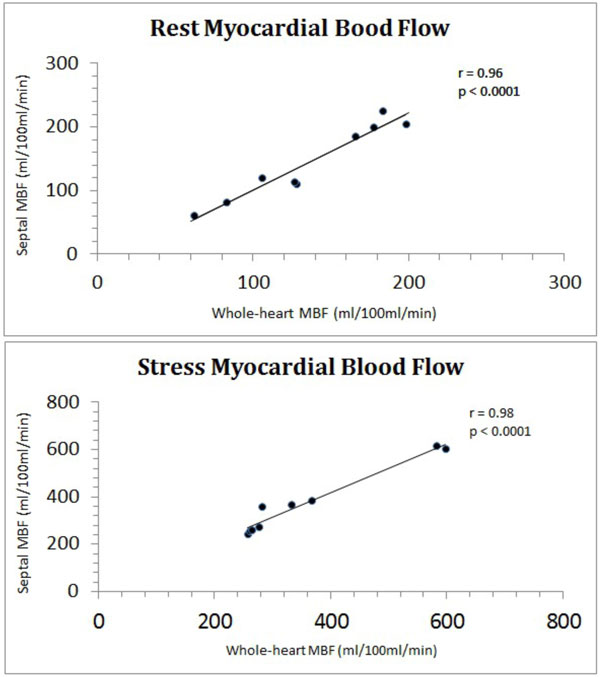
**Whole-heart versus septal myocardial blood flow in rest and stress**.

**Figure 2:**
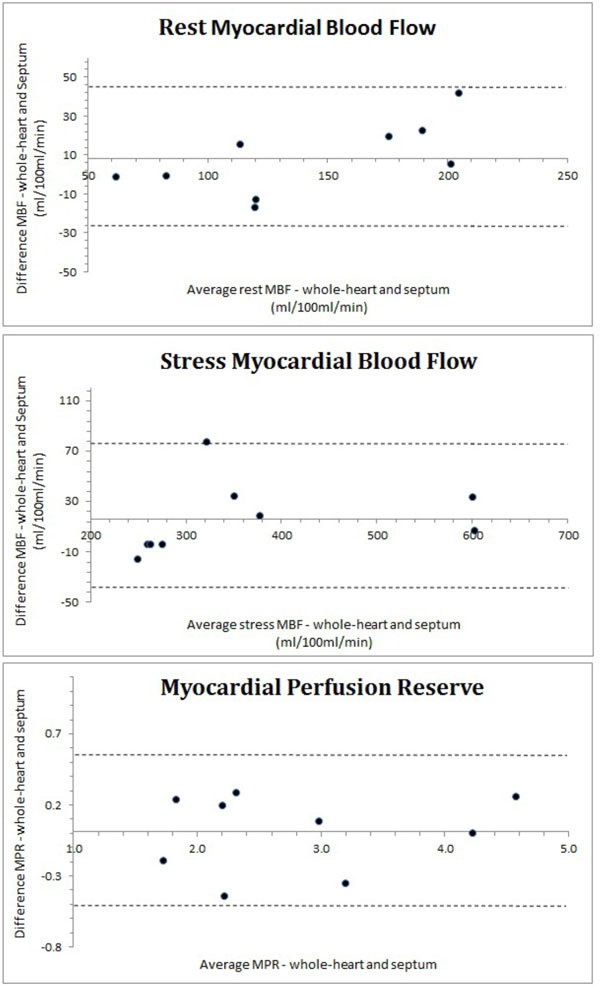
**Bland-Altman plots of myocardial blood flow, at rest and stress, and myocardial perfusion reserve**.

Mean age was 42 ± 11, 7 males (78%). All patients had adequate hemodynamic response. Whole-heart MBF estimation was 358 ± 137 ml/100 ml/min at stress and 137 ± 48 ml/100 ml/min at rest. Septal MBF was 374 ± 144 ml/100 ml/min at stress and 145 ± 60 at rest. Whole-heart MPR was 2.8 ± 1.02 and septal MPR was 2.81 ± 1.05. There was excellent agreement between whole-heart and septal MBF estimates at stress (r = 0.98; p < 0.0001) and rest (r = 0.96, p < 0.0001, Figure 1). Coefficient of variation between whole-heart and septal estimates for rest MBF, stress MBF and MPR were 8.2%, 6.7% and 7.8% respectively. Figure 2 shows Bland-Altman plots of MBF and MPR.

## Conclusions

Limited septal quantification of MBF is similar to whole-heart region of interest. This technique may simplify MBF estimation for those with suboptimal imaging outside of the septum or low myocardial signal.

## Funding

JPG and SP receive a research grant from Philips Healthcare. SP is funded by British Heart Foundation fellowship (FS/10/62/28409).

